# Capillary blood as an alternative specimen for enumeration of percentages of lymphocyte subsets

**DOI:** 10.1186/s13104-019-4659-4

**Published:** 2019-09-26

**Authors:** Supanart Srisala, Nutkridta Pongsakul, Thiantip Sahakijpicharn, Suradej Hongeng, Somchai Chutipongtanate, Nopporn Apiwattanakul

**Affiliations:** 1 Section for Research Center, Faculty of Medicine Ramathibodi Hospital, Mahidol University, 270 Rama VI Road, Bangkok, 10400 Thailand; 2 Department of Pediatrics, Faculty of Medicine Ramathibodi Hospital, Mahidol University, 270 Rama VI Road, Bangkok, 10400 Thailand

**Keywords:** Point-of-care, Lymphocyte subsets, Staining and labeling, Flow cytometry, Correlation study

## Abstract

**Objective:**

Capillary blood has been increasingly used in point-of-care setting for clinical monitoring in immunology and infectious diseases. We explored whether percentages of lymphocyte subsets (T-cells; CD3+, helper T-cells; CD4+, cytotoxic T-cells; CD8+, B-cells; CD19+, NK cells; CD56+, gamma delta T-cells, and regulatory T-cells) with regard to total lymphocyte count from capillary and venous blood of healthy volunteers were in good agreement.

**Results:**

All percentages of lymphocyte subsets with regard to total lymphocyte count from capillary blood were significantly correlated with those from venous blood (r ≥ 0.9 for every cell type). However, Bland–Altman plots showed high agreement between capillary and venous samples only in those of CD3+, CD4+, and CD8+ cells (limit of agreement percentages from mean venous blood < 20%). However, the agreement of percentages of other lymphocyte subsets from venous and capillary blood was mediocre. We concluded that capillary blood could be used as an alternative for venous blood to determine percentages of CD3+, CD4+, and CD8+ cells with regard to total lymphocyte count.

## Introduction

Lymphocyte subset enumeration by flow cytometry is widely used in the monitoring of many immunological and infectious diseases [1–4]. Patients with human immunodeficiency virus infection and those who have undergone stem cell transplantation require frequent lymphocyte subset enumerations to monitor the progression of diseases and to guide treatment [2, 5], hence, several venipuncture is required. Since venipuncture can be difficult in young infants or patients with fragile veins, therefore, it would be more convenient if capillary blood can be used. Capillary blood has been increasingly used as a point-of-care testing because less blood volume is needed and the technique used to obtain blood is simple [6]. A previous study found that numbers of T-cells (CD3+), helper T-cells (CD4+), cytotoxic T-cells (CD8+), B-cells (CD19+), and NK-cells (CD56+) from both venous and capillary blood were highly correlated [7]. However, discrepancy in the absolute numbers of some lymphocytes from venous and capillary samples were also noted [7, 8]. Furthermore, the numbers of regulatory T-cells (CD4+CD25+FoxP3+) and gamma delta (γδ) T-cells have not been compared using both techniques evaluated. Currently, these two lymphocyte subsets are increasingly recognized in immunology filed. Regulatory T-cells are involved in pathogenesis of several autoimmune diseases [9, 10], and sepsis [11]. Changes in γδ T-cells are associated with infection or the activity of inflammatory diseases [12–15]. If capillary blood is to be used for monitoring of these cells, it is essential to determine whether the numbers of these cells from capillary blood can well represent those from venous blood which is served as the current gold standard. In this study, we compared percentages of lymphocyte subsets; CD3+, CD4+, CD8+, CD19+, CD56+, CD3+CD56+ (NKT-cells), regulatory T-cells and γδ T-cells, with regard to total lymphocyte count in both venous and capillary blood samples from healthy adults. We also analyzed the agreement of these lymphocyte subset percentages between capillary and venous blood samples by using the well-established Bland–Altman method which is widely used to determine the agreement between two assays [16].

## Main text

### Subjects and blood samples

This study protocol was approved by the Research Ethical Committee of Ramathibodi Hospital, Mahidol University (ID 09-59-23), and subjects provided written informed consents. Forty healthy adult volunteers were enrolled. The health status of the volunteers was confirmed after complete blood counts were done and a medical examination by a medical doctor revealed that there was no inflammatory process in the volunteers. Non-fasting venous and capillary blood samples were collected from each volunteer. Venous blood were collected by venipuncture from the right cubital fossae, and capillary blood was collected from the tip of the right ring or middle fingers by a standard lancet (the OneTouch^®^ Lancing Device; Lifescan, Inc., CA, USA) with the first drop of blood being discarded to eliminate the contamination of tissue fluid [17]. Blood samples were collected into potassium ethylenediamine tetraacetate-containing tubes (BD Microtainer, Franklin Lakes, NJ, USA). The specimens were maintained at room temperature and were processed for immunophenotyping within 2 h.

### Immunophenotyping by flow cytometry

Lymphocyte subset phenotypes were determined by direct immunofluorescence staining with mononuclear antibodies (eBioscience Inc. San Diego, CA, USA) as following:

- Panel 1: anti-CD3-FITC (0.2 μg/μL), Anti-CD4-APC (0.012 μg/μL), anti-CD8-APC-efluor780 (0.025 μg/μL), anti-γδ TCR-PE (0.8 μg/μL), and anti-CD45-PECy7 (0.05 μg/μL).
- Panel 2: anti-CD3-FITC (0.2 μg/μL), anti-CD19-APC (0.025 μg/μL), anti-CD56-PE (0.025 μg/μL), and anti-CD45-PECy7 (0.05 μg/μL).
- Panel 3: anti-CD4-APC (0.012 μg/μL), anti-CD25-PECy7 (0.05 μg/μL).

Ten microliters of blood samples were incubated in darkness with the antibody (1:100 dilution) of each panel for 15 min at room temperature. Samples incubated with the antibody of panels 1 and 2 were then mixed with 100 μL of red blood cell lysing solution (Lysing Solution, BD Bioscience. San Jose, CA, USA) for 5 min at room temperature. The samples were then washed and were subjected to flow cytometry. Samples incubated with the antibody of panel 3 were fixed and permeabilized in 100 μL of Fixation/Permeabilization solution (eBioscience Inc. San Diego, CA, USA) for 30 min at room temperature. Samples were then washed with permeabilization buffer (eBioscience Inc. San Diego, CA, USA), and incubated in 25 μL of permeabilization buffer containing 1:50 dilution of Anti-Foxp3-FITC (0.1 μg/μL) for 45 min at room temperature. After being washed, the samples were subjected to flow cytometry (BD FACSVerse; BD Biosciences, San Jose, CA, USA, 2012).

Cells were analyzed by using Flowjo software version 10 (Flowjo, LLC; Ashland, OR, USA). The gating strategy for each cell type is summarized in Additional file 1: Table S1. Since our study did not use a single platform approach, absolute cell count for each lymphocyte subset is not available, and only the percentages of lymphocyte subsets with regard to total lymphocyte count will be presented.

### Statistical analysis

Statistical analysis was performed with SPSS version 20 (SPSS, Inc., Chicago, IL, USA). Data were first tested with Shapiro–Wilk test for normality. The differences of percentages of lymphocyte subsets with regard to total lymphocyte count between capillary and venous blood with normal distribution were analyzed by 2-tailed paired t-test and those without normal distribution were analyzed by Wilcoxon Signed Ranks. The type I error rate (alpha level) of < 0.05 is set as statistical significance. Correlations between percentages of lymphocyte subsets from venous and capillary blood were analyzed by Pearson correlation. To determine agreement of percentages of lymphocyte subsets from venous and capillary blood, Bland–Altman analyses were performed. Differences between each pair of measurements (capillary value − venous value) were plotted on the vertical axis against the averages of the pair (capillary value + venous value)/2 on the horizontal axis [16]. Bias was the difference of results between capillary and venous samples. Limit of agreement (LOA) was within two standard deviations from the mean bias [16].

### Results

**Fig. 1:**
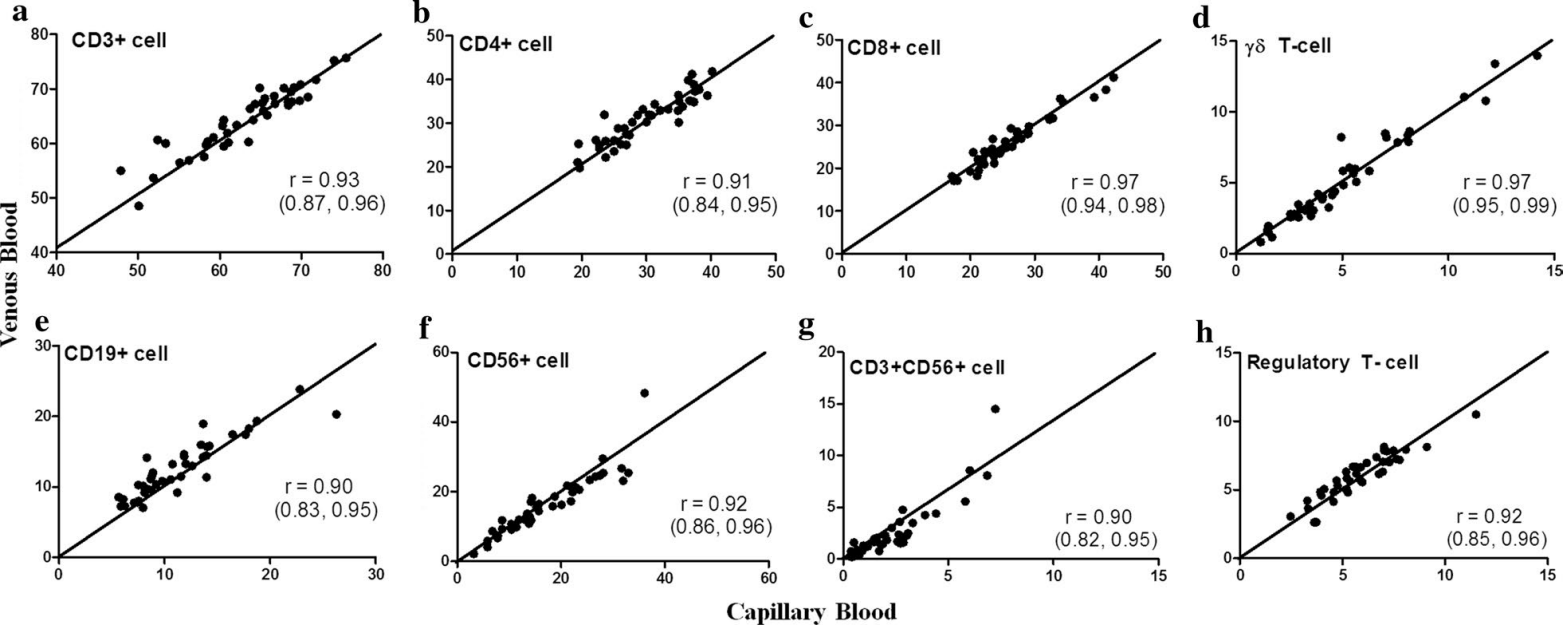
Correlations of percentages of lymphocyte subsets between venous and capillary blood. Pearson correlation analyses of percentages of lymphocyte subsets from venous and capillary blood were performed; **a** CD3+ cells, **b** CD4+ cells, **c** CD8+ cells, **d** γδ TCR+ cells, **e** CD3−CD56+ (NK−) cells, **f** CD3+CD56+ (NKT−) cells, **g** CD19+ (B−) cells, and **h** CD4+CD25+FoxP3+ (Treg) cells. r represents Pearson r value. 95% confidence intervals are provided in the parentheses. The correlations of all cell types were statistically significant difference (P < 0.05)

Among the 40 healthy adult subjects, 27 (67.5%) were female. Their ages ranged from 26 to 57 years, with a median value of 31 years. The percentages of lymphocyte subsets with regard to total lymphocyte count collected from capillary and venous blood are shown in Additional file 2: Table S2). The percentages of CD3+, CD4+, and CD19+ cells from venous blood were slightly higher and that of CD56+ was slightly lower than those from capillary blood, the magnitudes of these differences were < 10% between each other. The percentages of other cell subsets did not significantly differ between venous and capillary blood. Pearson correlation coefficients for the percentages of all lymphocyte subsets from capillary and venous blood were all > 0.8 (Fig. 1a–h).

**Fig. 2:**
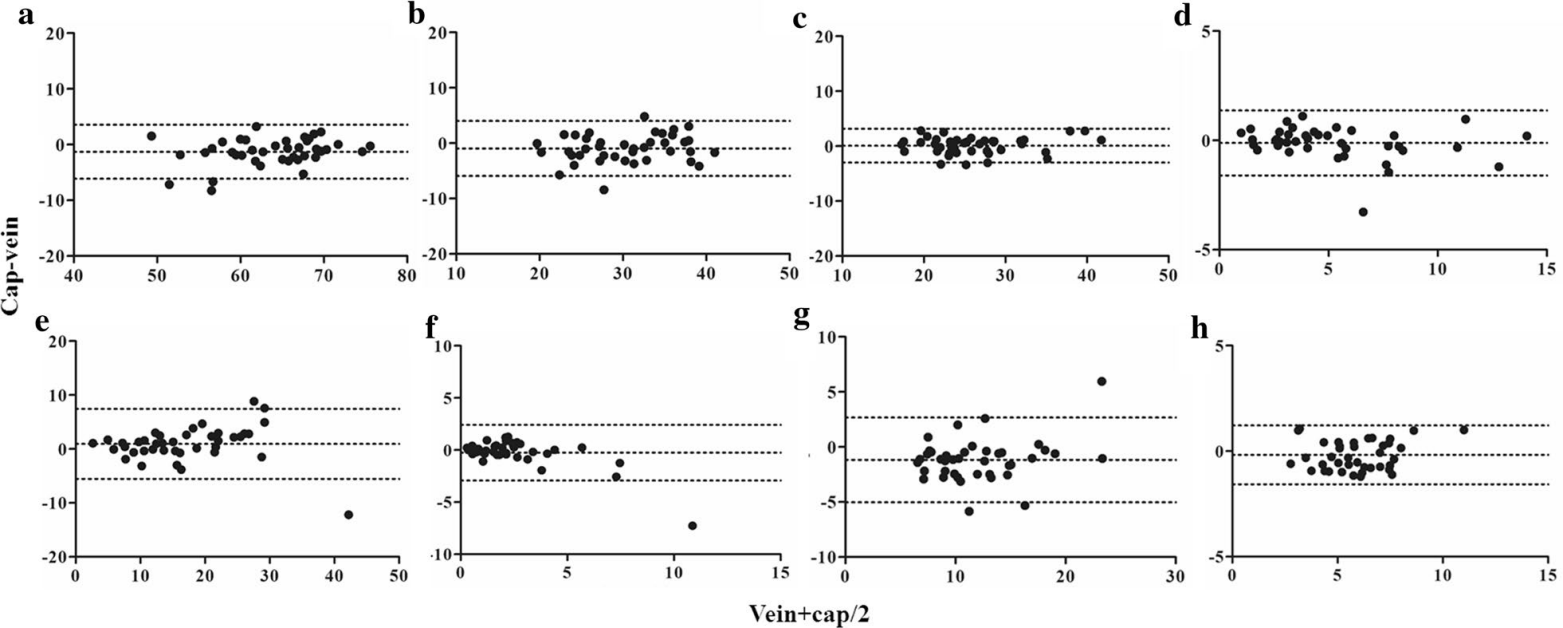
Bland–Altman plots for agreement of percentages of lymphocyte subsets between venous and capillary blood. Bland–Altman analyses of percentages of lymphocyte subsets from venous and capillary blood were performed; **a** CD3+ cells, **b** CD4+ cells, **c** CD8+ cells, **d** γδ TCR+ cells, **e** CD3−CD56+ (NK−) cells, **f** CD3+CD56+ (NKT−) cells, **g** CD19+ (B−) cells, and **h** CD4+CD25+FoxP3+ (Treg) cells. Middle dot lines represent mean bias values. Upper and lower dot lines represent 95% confidence intervals of upper and lower limits of agreement, respectively

Bland–Altman bias plots were constructed by plotting the differences between the percentages of cells from venous and capillary blood against the mean percentage values of the two methods (Fig. 2). Table 1 shows the Bland–Altman parameters, including ranges, bias values, and limits of agreement (LOA). The percentages of LOA from mean venous values of CD3+, CD4+, CD8+ were < 20%, while those of γδ T-cells, regulatory T-cells, CD19+, and CD56+ cells were in the range of 20–40%. The LOA of CD3+CD56+ cells showed the highest value, exceeding 100%. Individual data of percentages of lymphocyte subset are provided in Additional file 3 and parameters derived from Bland Altman analysis of each lymphocyte subset are provided in Additional file 4.

**Table 1:** Bland–Altman parameters for agreement of percentages of lymphocyte and lymphocyte subsets between venous and capillary blood

Cells	Max value	Min value	Mean venous blood	Bias ± SD	Bias percentage from mean venous blood	95% LOA	LOA percentage from mean venous blood
Lymphocytes	46.34	11.99	27.64	− 0.13 ± 3.46	0.48	6.64, − 6.91	24.52
CD3+	75.69	48.54	64.40	− 1.27 ± 2.47	1.97	3.57, − 6.11	7.52
CD4+	41.86	19.74	30.87	− 0.96 ± 2.53	3.10	4.01, − 5.92	16.08
CD8+	41.22	17.08	26.07	0.10 ± 1.57	0.38	3.18, − 2.98	11.80
γδ TCR+	13.98	0.82	5.23	− 0.11 ± 0.76	2.15	1.37, − 1.60	28.35
CD3−CD56+	33.81	1.93	14.05	1.19 ± 2.59	8.48	6.26, − 3.87	36.06
CD3+CD56+	14.5	0.18	2.56	− 0.25 ± 1.37	9.77	2.43, − 2.93	104.71
CD19+	23.87	7.07	12.62	− 1.17 ± 1.97	9.27	2.68, − 5.02	30.53
CD4+CD25+Foxp3+	10.5	2.63	5.93	− 0.17 ± 0.71	2.93	1.22, − 1.57	23.47

*SD* standard deviation, *LOA* limit of agreement, *TCR* T cell receptor

### Discussion

In this study, the percentages of lymphocyte subsets with regard to total lymphocyte count from capillary and venous blood were comparable and highly correlated. Although there were statistically significant differences in CD3+, CD4+, CD19+, and CD56+ cells, the magnitudes of differences were sufficiently subtle (< 10%) that they are unlikely to exhibit any clinical significance. We further analyzed this agreement by using the Bland–Altman method. Currently, the appropriate cut-off LOA percentages from mean venous blood of lymphocyte subsets to indicate that the results from capillary and venous blood are in good agreement if the LOA values calculated are below these cut-off values are still not known. The ranges of acceptable LOA may be extrapolated from some closely related studies. The Clinical Laboratory Improvement Amendments of 1988 (CLIA-88) regulations designated that total white blood cell count should not fall outside 15% of the reference value [19]. Hollis et al. [18] found that the LOA percentages from the mean venous values of total white blood cell, granulocyte, and lymphocyte, were all < 20%, while those of monocyte count was 40%. In our study, LOA percentages from the mean venous blood of CD3+ and CD8+ cells were < 15%; that of CD4+ cells was 16%. This was quite similar to the study of Sitoe et al. [20] who reported the LOA percentage of CD4+ cells from mean venous blood of 18%. Our study suggests that capillary blood is a good alternative for venous blood to determine the percentages of CD3+, CD4+, and CD8+ cells with regard to total lymphocyte count, based on the fact that the LOA percentages of these cells were quite low. However, judicious use of capillary blood for monitoring of γδ T-cells, regulatory T-cells, NK-cells, and B-cells is suggested, as the LOA percentages of these cells were quite high. Capillary blood should not be used for monitoring of NKT cells because the LOA percentage of this cell was very high (LOA 104%).

In previous studies, the results of agreement of cell enumeration between capillary and venous blood were varied. Yang et al. [8] demonstrated that the total and large leukocyte counts were significantly higher in capillary than in venous blood. Cracknell et al. [7] found that the numbers of most lymphocyte subsets were higher in capillary blood. However, these studies did not use Bland–Altman method to evaluate the agreement. Furthermore, there have been no studies showing the agreement of percentages of γδ T-cells and regulatory T-cells between capillary and venous blood. This study showed that the percentages of these two cell subsets did not differ between the two sources and the correlation coefficients of the percentages of these 2 cells between venous and capillary blood were > 0.9. However, the percentages of LOA from Bland–Altman bias plots for these 2 cells were slightly high when compared with those of CD3+, CD4+, and CD8+ cells; 28 and 23%, respectively. This emphasizes that if only the correlation coefficients were considered, the results could be misleading. This underscores the importance of using Bland–Altman bias plots to evaluate agreement of a new method with the standard method. Several reasons may explain the discrepancies in the percentages of lymphocyte subsets between the two sources. Squeezing the fingertip before puncture [8, 21] or the presence of tissue injury, which can induce adhesion of lymphocytes to capillary bed [22], may contribute to this discrepancy. This emphasizes the importance of practicing good techniques in obtaining capillary blood samples. Injury from fingertip puncture should be as minimal as possible by using appropriate lancets. Good blood flow after puncture should be obtained to minimize the need of squeezing.

The strength of our study is that we used Bland–Altman analysis to determine the agreement of percentages of lymphocyte subsets from venous and capillary blood. To our knowledge, apart from percentage of CD4+ T-cells [20], the agreement of percentages of other lymphocyte subsets has not been previously reported. Our results showed that capillary blood could be used as an alternative for venous blood to determine percentages of CD3+, CD4+, and CD8+ cells with regard to total lymphocyte count. These data will support point-of-care assays using capillary blood as samples [23, 24]. Because mobile flow cytometers are currently available [25], samples from capillary blood should facilitate point-of-care testing purposes for enumeration of CD3+, CD4+, and CD8+ cells which are frequently monitored in many diseases. However, additional studies are warranted before capillary blood can accurately be used as an alternative for venous blood for enumeration of percentages of γδ T-cells, NK-cells, B-cells, and regulatory T-cells as the agreement of the percentages of these cells with regard to total lymphocyte count is still mediocre.

## Limitations

- Study was performed within a small number of subjects, and repeated measurements were not performed.
- Study did not include various subject categorizations based on age, sex, and underlying hematological or immunological diseases.
- Absolute lymphocyte count could not obtain from this study as we did not use a single platform approach. Complete blood count is needed to enumerate the total count of each lymphocyte subset.

Application of capillary blood for lymphocyte subset enumeration with mobile flow cytometer as a point-of-care setting is promising but needs further validation before implementation.

## Supplementary information

**Additional file 1: Table S1.** Gating strategies for all lymphocyte subsets. All cells were initially gated from the CD45+ population, and then gated from the lymphocyte population, as determined by FSC/SSC plot.

**Additional file 2: Table S2.** Head to head comparison of median and mean percentages of lymphocyte subsets with regard to total lymphocyte count in venous and capillary blood (n = 40 for both capillary and venous blood samples).

**Additional file 3.** Individual data of percentage of each lymphocyte subset with regard to total lymphocyte count both from capillary and venous blood samples.

**Additional file 4.** Summary of data from Bland Altman analysis.

## Data Availability

All data generated or analysed during this study are included in this published article in Additional files.

## Abbreviations

CD3+: T-cells
CD4+: helper T-cells
CD8+: cytotoxic T-cells
CD19+: B-cells
CD56+: NK-cells (natural killer cells)
CD3+CD56+: NKT-cells (natural killer T-cells)
CD4+CD25+FoxP3+: regulatory T-cells
γδ T-cells: gamma delta T-cells
LOA: limit of agreement (in Bland–Altman analysis); within two standard deviations from the mean bias (bias was the difference of results between capillary and venous samples)
CLIA-1988: Clinical Laboratory Improvement Amendments of 1988

## References

[1] Abdallah KO, Prak ET. B cell monitoring of transplant patients treated with anti-CD20. Clin Transpl. 2006:427–37. 18365400

[2] Hoare RL, Veys P, Klein N, Callard R, Standing JF (2017). Predicting CD4 T-cell reconstitution following pediatric hematopoietic stem cell transplantation. Clin Pharmacol Ther.

[3] Hogg RS, Yip B, Chan KJ (2001). Rates of disease progression by baseline CD4 cell count and viral load after initiating triple-drug therapy. JAMA.

[4] Siedner MJ, Ng CK, Bassett IV, Katz IT, Bangsberg DR, Tsai AC (2015). Trends in CD4 count at presentation to care and treatment initiation in sub-Saharan Africa, 2002–2013: a meta-analysis. Clin Infect Dis.

[5] Mermin J, Ekwaru JP, Were W (2011). Utility of routine viral load, CD4 cell count, and clinical monitoring among adults with HIV receiving antiretroviral therapy in Uganda: randomised trial. BMJ.

[6] St John A, Price CP (2014). Existing and emerging technologies for point-of-care testing. Clin Biochem Rev.

[7] Cracknell SE, Hinchliffe RF, Lilleyman JS (1995). Lymphocyte subset counts in skin puncture and venous blood compared. J Clin Pathol.

[8] Yang ZW, Yang SH, Chen L, Qu J, Zhu J, Tang Z (2001). Comparison of blood counts in venous, fingertip and arterial blood and their measurement variation. Clin Lab Haematol.

[9] Giang S, La Cava A (2016). Regulatory T cells in SLE: biology and use in treatment. Curr Rheumatol Rep.

[10] Ohl K, Tenbrock K (2015). Regulatory T cells in systemic lupus erythematosus. Eur J Immunol.

[11] Cao C, Ma T, Chai YF, Shou ST (2015). The role of regulatory T cells in immune dysfunction during sepsis. World J Emerg Med.

[12] Andreu-Ballester JC, Tormo-Calandin C, Garcia-Ballesteros C (2013). Association of gammadelta T cells with disease severity and mortality in septic patients. Clin Vaccine Immunol.

[13] Perko R, Kang G, Sunkara A, Leung W, Thomas PG, Dallas MH (2015). Gamma delta T cell reconstitution is associated with fewer infections and improved event-free survival after hematopoietic stem cell transplantation for pediatric leukemia. Biol Blood Marrow Transplant.

[14] Venet F, Bohe J, Debard AL, Bienvenu J, Lepape A, Monneret G (2005). Both percentage of gammadelta T lymphocytes and CD3 expression are reduced during septic shock. Crit Care Med.

[15] Lertbunrian R, Chonpaisan K, Srisala S, Anurathapan U, Apiwattanakul N (2019). Decrease in gamma delta T-cell with microbiologically proven infection in septic oncologic children. J Med Assoc Thai.

[16] Bland JM, Altman DG (1986). Statistical methods for assessing agreement between two methods of clinical measurement. Lancet.

[17] MacLennan CA, van Oosterhout JJ, White SA, Drayson MT, Zijlstra EE, Molyneux ME (2007). Finger-prick blood samples can be used interchangeably with venous samples for CD4 cell counting indicating their potential for use in CD4 rapid tests. AIDS.

[18] Hollis VS, Holloway JA, Harris S, Spencer D, van Berkel C, Morgan H (2012). Comparison of venous and capillary differential leukocyte counts using a standard hematology analyzer and a novel microfluidic impedance cytometer. PLoS ONE.

[19] Medicare, Medicaid and CLIA programs; regulations implementing the Clinical Laboratory Improvement Amendments of 1988 (CLIA)-HCFA. Final rule with comment period. Fed Regist. 1992;57:7002–186. 10170937

[20] Sitoe N, Luecke E, Tembe N (2011). Absolute and percent CD4+ T-cell enumeration by flow cytometry using capillary blood. J Immunol Methods.

[21] Daae LN, Halvorsen S, Mathisen PM, Mironska K (1988). A comparison between haematological parameters in ‘capillary’ and venous blood from healthy adults. Scand J Clin Lab Invest.

[22] Cavender DE (1989). Lymphocyte adhesion to endothelial cells in vitro: models for the study of normal lymphocyte recirculation and lymphocyte emigration into chronic inflammatory lesions. J Invest Dermatol.

[23] Jani IV, Sitoe NE, Chongo PL (2011). Accurate CD4 T-cell enumeration and antiretroviral drug toxicity monitoring in primary healthcare clinics using point-of-care testing. AIDS.

[24] Mtapuri-Zinyowera S, Chideme M, Mangwanya D (2010). Evaluation of the PIMA point-of-care CD4 analyzer in VCT clinics in Zimbabwe. J Acquir Immune Defic Syndr.

[25] Balsam J, Bruck HA, Rasooly A (2015). Mobile flow cytometer for mHealth. Methods Mol Biol.

