# Supplementary material for: Capillary blood as an alternative specimen for enumeration of percentages of lymphocyte subsets
Source: BMC Res Notes. 2019 Sep 26;12:633. doi: 10.1186/s13104-019-4659-4 (PMC6761722; doi:10.1186/s13104-019-4659-4)
Supplement: Supplementary file 1 — Additional file 1: Table S1. Gating strategies for all lymphocyte subsets. All cells were initially gated from the CD45+ population, and then gated from the lymphocyte population, as determined by FSC/SSC plot. [file 13104_2019_4659_MOESM1_ESM.docx]

**Table S1** Gating strategies for all lymphocyte subsets. All cells were initially gated from the CD45+ population, and then gated from the lymphocyte population, as determined by FSC/SSC plot.

| **Lymphocyte subsets** | **Gating strategy** |
| --- | --- |
| CD3+ cells | CD45+ CD3+ |
| CD4+ T-cells | CD45+ CD3+ CD4+ |
| CD8+ T-cells | CD45+ CD3+ CD8+ |
| Regulatory T-cells (Treg) | CD4+ CD25+ FoxP3+ |
| B-cells | CD45+ CD3- CD19+ |
| NK-cells | CD45+ CD3- CD56+ |
| NKT-cells | CD45+ CD3+ CD56+ |
| γδ T-cells | CD45+ CD3+ CD4- CD8- γδ TCR+ |
