# Supplementary material for: Capillary blood as an alternative specimen for enumeration of percentages of lymphocyte subsets
Source: BMC Res Notes. 2019 Sep 26;12:633. doi: 10.1186/s13104-019-4659-4 (PMC6761722; doi:10.1186/s13104-019-4659-4)
Supplement: Supplementary file 2 — Additional file 2: Table S2. Head to head comparison of median and mean percentages of lymphocyte subsets with regard to total lymphocyte count in venous and capillary blood (n = 40 for both capillary and venous blood samples). [file 13104_2019_4659_MOESM2_ESM.docx]

**Table S2** Head to head comparison of median and mean percentages of lymphocyte subsets with regard to total lymphocyte count in venous and capillary blood# (n=40 for both capillary and venous blood samples)

**Lymphocyte subsets Capillary blood Venous blood P-value**

Lymphocyte/total WBC 27.23 (23.35, 32.83) 27.32 (22.03, 32.00)

27.55 (6.72) 27.64 (7.22) 0.893*

(25.40, 29.70) (25.34, 29.95)

CD3+ cells 64.20 (58.69, 68.37) 65.58 (60.22, 68.44)

63.12 (6.59) 64.40 (5.90) 0.002*

(61.02, 65.23) (62.51, 66.28)

CD3+ CD4+ cells 29.75 (25.02, 35.48) 31.80 (25.93, 34.96)

29.91 (6.11) 30.87 (5.66) 0.022*

(27.96, 31.87) (29.06, 32.68)

CD3+ CD8+ cells 24.91 (21.96, 29.01) 24.78 (22.09, 29.15) 0.610^+^

26.17 (6.08) 26.07 (5.89)

(4.13, 6.11) (4.19, 6.28)

γδ TCR+ cells 4.45 (2.94, 6.84) 4.17 (2.79, 7.89) 0.677^+^

5.12 (3.09) 5.23 (3.27)

(4.13, 6.11) (4.19, 6.28)

CD3-CD56+ cells 13.67 (8.62, 20.91) 13.45 (8.52, 18.96) 0.0058^*^

15.24 (7.72) 14.05 (7.04)

(12.77, 17.71) (11.80, 16.30)

CD3+ CD56+ cells 1.96 (0.90, 2.85) 1.76 (0.91, 2.87) 0.677^+^

2.31 (1.75) 2.56 (2.71)

(1.75, 2.87) (1.69, 3.42)

CD19+ cells 10.67 (8.00, 13.88) 11.52 (9.30, 15.44) 0.000^+^

11.45 (4.61) 12.62 (4.11)

(9.97, 12.92) (11.30, 13.93)

CD4+CD25+ Foxp3+ cells 5.58 (4.58, 6.99) 6.05 (4.86, 7.09)

5.76 (1.79) 5.93 (1.74) 0.130*

(5.19, 6.33) (5.38, 6.49)

^#^Data in the first, second, and third rows of each parameter represent median and interquartile range (IQR), mean and standard deviation (SD), and 95% confidence interval, respectively.

*paired sample t-test analysis, +paired sample Wilcoxon Signed Ranks analysis. P-value <0.05 is considered statistically significant.

Abbreviations: WBC, white blood cell
